# Utilization of medicinal hemp: a qualitative analysis of clinicians’ perspectives in Ghana

**DOI:** 10.1186/s42238-025-00271-1

**Published:** 2025-03-17

**Authors:** Radolf Ansbert Nortey, Anoa Aidoo, Seth Kwabena Amponsah

**Affiliations:** 1 Department of Pharmacy Practice, Faculty of Pharmacy and Pharmaceutical Sciences, Kwame Nkrumah University of Science and Technology (KNUST), PMB, Kumasi, Ghana; 2 Department of Pharmacy Practice and Clinical Pharmacy, University of Ghana School of Pharmacy, Accra, P.O. Box LG 43 Legon, Ghana; 3 Department of Pharmacology, University of Ghana Medical School, Accra, P.O. Box LG 43, Ghana

**Keywords:** Medicinal hemp, Doctors, Cannabinoids, Therapeutic, Legislation, Africa, Ghana

## Abstract

**Background:**

Previous studies have explored the perspectives of health care professionals on the use of medicinal hemp, but they were mostly situated in high income countries. This study sets out to investigate the knowledge, attitudes, and beliefs of health care professionals regarding the use of hemp in clinical therapy vis-à-vis the legislative framework in Ghana.

**Objective:**

To explore the perspectives of physicians on the use of medicinal hemp and its legalization in Ghana.

**Method:**

A qualitative study employing purposive sampling and face-to-face in-depth interviews was conducted among twenty (20) physicians practicing in Ghana. The interviewees were selected based on specialty and scope of practice. The in-depth interviews were audio recorded, transcribed, and thematically analyzed using the Theory of Planned Behavior.

**Results:**

Twenty (20) clinicians participated in the study. Fourteen (14) respondents were male and six (6) were female. The medical specialties of the interviewees were anaesthesiology, oncology, neurology, and orthopaedics. The identified themes include clinical use, stigma (sociocultural classification of cannabis), clinical non-prescription, policy landscape and regulatory control.

**Conclusion:**

Prescribers’ intention to recommend medicinal hemp is influenced by a complex interplay of various socio-political factors such as knowledge, policy, cultural environment, and stigma.

**Supplementary Information:**

The online version contains supplementary material available at 10.1186/s42238-025-00271-1.

## Background

The 1961 United Nations Single Convention on Narcotic Drugs defines cannabis or hemp as “the flowering or fruiting tops of the cannabis plant from which the resin has not been extracted, by whatever name they may be designated in commerce” (UNODC 1961; United Nations Office on Drugs and Crime 1950). Medicinal cannabis or hemp refers to *Cannabis sativa* or *Cannabis indica* plants used to treat diseases or relieve symptoms of a medical condition (Caulkins et al. 2012).

There has been substantial research on the perspectives of health care professionals considering the vital role they play in medical policies on cannabis (Gardiner et al.^2019^; Rønne et al. 2024). Alexandra et al. studied the perspectives and knowledge of physicians on medical marijuana in Washington (Sideris et al. 2024). Zolotov et al. also studied the perceptions of Israeli physicians and queried medical cannabis as an oxymoron (Zolotov et al. 2018). Recent systematic review analysis of physicians’ perspectives on medical cannabis reported both support for the therapeutic potential of cannabis and concern about indirect societal harm (Gardiner et al.^2019^; Rønne et al. 2024). Despite the available research, these studies exist mainly in developed settings such as Canada, the USA, and Israel (Rønne et al. 2024; Erku et al. 2024). Research is lacking in developing countries such as those situated in Africa (Kitchen et al. 2024a). The knowledge, attitudes, and beliefs of health care professionals regarding medicinal hemp or cannabis in Ghana is yet to be explored. With the recent shift in global policies on cannabis, various jurisdictions are permitting legal access to cannabis (Rønne et al. 2024). The discussions are no different in Ghana especially with African countries such as Zimbabwe and Uganda joining a long list of countries worldwide (Ghanaweb. General News. 2020).

Currently, in Ghana, the cultivation and use of hemp for medicinal and industrial use is permitted under strict regulations (Parliament of Ghana 2020). The caveat is that the tetrahydrocannabinol (THC) component should not exceed 0.3% w/v and a license should be issued to that effect (Parliament of Ghana 2020) The Narcotics Control Commission (Cultivation and Management of Cannabis) Regulations, 2023 (L.I. 2475) empowers the Minister of Interior to grant licenses for hemp cultivation and outlines the operational framework for import and export (Republic of Ghana. Narcotics Control Commission (Cultivation and Management of Cannabis) Regulations. Ghana Gazette, L.I 2475 Ghana : Parliament of Ghana 2475; (Agyemang Hanson and Newsroom. 2023; Ackah-Blay and Myjoyonline. News. 2023)). Locally, there is considerable commercial interest in the cultivation of hemp and its positive economic potential through export (AfricaNews and Parliament passes NACOC Bill to allow cannabis cultivation for industrial and medicinal purposes | Ghana. 2023), (Quansah 2024).

Common to the general controversy surrounding cannabis use, societal concerns are associated with the possible adverse health effects and health professionals are at the forefront of this opinion Table (Tetteh et al. 2012; Ghana and News. 2022; World Health Organization. Programme on Substance Abuse: Approaches to Treatment of Substance Abuse.^1993^.Available from:https://www.who.int/docs/default-source/substance-use/who-psa-93-10.pdf.cited^2024^ Jan 25,1993; Gardiner et al. 2023). In Ghana, the major viewpoint of the medical community reflects the opinion of psychiatrists and other mental health professionals communicating the adverse psychotic effects of cannabis (Quansah 2024), (Ghana and News. 2022), (Ghana and News. 2017).

Given the reported diverse therapeutic potential of cannabis and the need for a broader consultation among healthcare professionals (Gardiner et al. 2023), (Leinen et al. 2023), this research aims to investigate the knowledge, attitudes, and beliefs of health care professionals regarding the role of medicinal hemp in clinical therapy and its legislation in Ghana. The outcomes of this study aim to aid stakeholder agencies such as the Ministry of Health in making decisions regarding future policies on medicinal hemp.

## Method

### Study design

We conducted a qualitative cross-sectional study. This involved the use of in-depth interviews with open-ended questions to enable the exploration of new ideas and deeper probing into the physician’s opinions on medicinal hemp. The interview protocol was developed on the basis of the available literature on medicinal hemp or cannabis and was structured to examine the knowledge, attitudes, and perspectives of physicians within the theoretical construct of the Theory Planned Behavior (Table 1).

**Table 1:** The relationship between interview questions and the theory of planned behaviour framework

Theory of Planned Behaviour Variable	Interview Question
^1^ Attitude toward behaviour *Attitudes toward medical cannabis prescribing and use can be described as a result of one’s technical knowledge and personal opinions about the therapeutic potential or role of medical cannabis in clinical practice*	• In your opinion what is medical cannabis? • Are there disease conditions requiring the use of medical cannabis? • Can you tell me about the clinical efficacy of cannabis for the aforementioned indications?
^2^ Subjective norm *Clinicians’ perceptions of what significant parties think about the use of medicinal cannabis in clinical therapy. It accounts for the individual’s impression on the opinions of others such as colleagues, superiors, patients *etc. *on the utilization of medical cannabis*	• Are there any alternative treatment options aside cannabis in these instances; and how do they compare with cannabis in terms of efficacy? • Are there any clinical guidelines or policies outlining medicinal cannabis use in Ghana? (if yes, which?)
^3^ Perceived behavioural control *Clinicians’ beliefs about the degree of control they possess about the decision to prescribe or recommend medical cannabis in clinical care*	• Has the recent legalization of medicinal cannabis affected clinical practice in Ghana? • Has legalization of cannabis affected acceptance of its use?
^4^ Behavioural intention *The cumulative effect of the attitudes, subjective norms, and perceived behavioural control. It can be described as the sum of motivational factors that influences a clinician or doctor to recommend medicinal cannabis to a patient*	• Is access and use medical cannabis necessary for your practice as a doctor? • Considering the scope of medical practice in Ghana, is there a need to include medicinal cannabis as part of therapy?

**Fig. 1:**
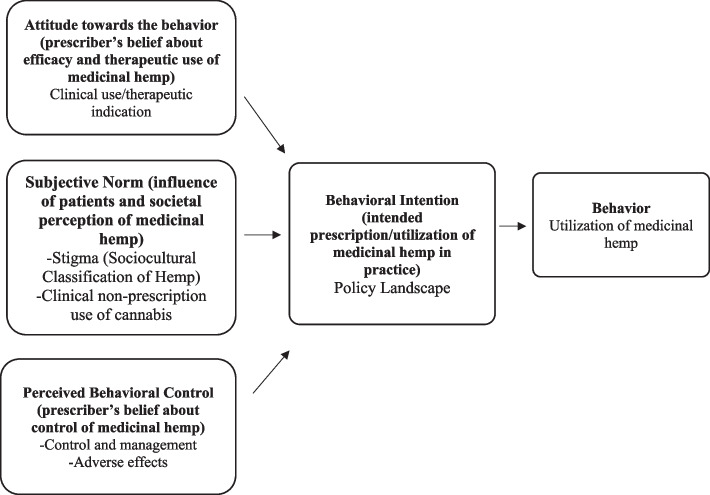
Application of the theory of planned behavior model in utilization of medicinal hemp

The TPB offers a theoretical approach to understanding physicians’ behavior and the intention to prescribe (Liu et al. 2024; Wash et al. 2024; Rashidian and Russell 2024)). The TPB framework postulates volitional human behavior as an aggregation of ‘attitudes toward the behavior’, ‘subjective norms’, and ‘perceived behavioral control’ (Fig. 1) (Kan and Fabrigar 2024), (Sniehotta et al. 2024).

### Study setting & population

The study was conducted in Accra, Ghana. Accra is the capital of Ghana and has a good number of specialist clinics or hospitals. The respondents were purposively selected from specialist clinics or hospitals that provide oncology, orthopedic, pain management or neurology care to patients. Eligible study participants were medical doctors who were practicing in Ghana at the time of the study. The lead researcher (RN) sent a request letter to the medical director of the respective institution, who then provided the contact information of the required specialists, a research focal person or a doctor who could assist with further introduction to their colleagues. The research team compiled a list of 44 potential study participants. The researchers attempted to reach all the clinicians by phone or email to book an interview appointment. Twenty-seven doctors agreed to participate, 4 doctors did not respond to the phone call/texts, and 3 doctors requested for a call but refused to answer the follow up calls/texts from the research team. The remaining 10 individuals refused to participate in the study. Among the 27 participants who agreed, 20 doctors were successfully contacted in-person for the study interviews.

### Inclusion criteria

Specialists or residents in oncology, orthopedics, pain medicine, neurology, and family medicine were recruited for the study. These specialties were pre-selected to represent clinicians who were likely to find an indication for medicinal hemp in their practice. Only physicians who were willing to participate in the study were included.

### Exclusion criteria

Medical practitioners who were not licensed to practice in Ghana.

### Population Sampling

Purposive sampling was employed for this study. Twenty (20) physicians were recruited for this study. The sample size determination was guided by the quality and relevance of data collected, where gathering new data no longer unearthed any new theoretical insights (Vasileiou et al. 2024).

### Data collection and management

Data were collected via face-to-face semi-structured interviews using a pre-designed interview guide. The interview guide explored thematic areas related to personal information, general knowledge on cannabis/hemp, medicinal hemp use in Ghana, and knowledge of legislative structures**.** The interviews were audio-recorded with the consent of the participants and transcribed for analysis.

### Quality control

The interview guide was pretested with five (5) medical practitioners to ensure the reliability and validity of the data collection tool.

### Data analysis

The data from the interviews were categorized and grouped into themes after transcription. The framework of the thematic analyses was as espoused by Fishbein and Ajzen (Trafimow 2009), and is based on the theory of planned behavior (Kan and Fabrigar 2024). The theory of planned behavior is a social psychological model that explains how people make decisions. It posits that people’s intentions to perform a behavior are determined by their attitudes toward the behavior and their subjective norms about the perceived behavioral control (Kan and Fabrigar 2024). The thematic reporting will employ the use of descriptive tables alongside direct quotes from the interviewees.

### Ethical consideration

Ethical approval was obtained from the University of Ghana School of Pharmacy Ethics Review Committee. The participants provided written consent for their involvement in the study after being made aware of the objectives. Participation was based on willingness and responses were kept anonymous to protect the privacy and confidentiality of the interviewees.

## Results

A total of 20 medical doctors were engaged on their perspectives on the utilization and regulation of cannabis within the healthcare setting (Tables 2 and 3).

**Table 2:** Summary characteristics of 20 interviewed clinicians

Variable	Number	Percentage (%)
*Gender*		
Male	**14**	**70**
Female	**6**	**30**
*Age range (years)*		
< 25	**-**	**-**
25—35	**6**	**30**
36—45	**10**	**50**
45—55	**4**	**20**
> 55	**-**	**-**
*Country of Medical Training*		
Ghana	**19**	**95**
Abroad (Outside Ghana)	**1**	**5**
*Specialty*		
Anaesthesiology	**4**	**20**
Oncology	**5**	**25**
Neurology	**6**	**30**
Orthopaedic	**5**	**25**
*Type of Institution*		
Government	**14**	**70**
Private	**6**	**30**
*Number of years in practice (years)*		
< 5	**-**	**-**
5 – 10	**12**	**60**
11 – 20	**8**	**40**
> 20	**-**	**-**

**Table 3:** Themes and subthemes matched with theory of planned behavior components

Theory component	Theme	Subtheme
**Attitudes**	Clinical use/ indication	Knowledge on medicinal hemp Clinical efficacy of medicinal hemp supported by scientific evidence Therapeutic application of medicinal hemp
*“Its use is not so common in our practice so most of the things I am telling you is based on literature…in mainstream neurology according to literature Medicinal cannabis can be used for refractory seizures and pain management, it could be oncology, neuropathic pain, it could be sickle cell.”* *“They will say they have the oil and they will swear by it that when they take it, it really helps to control their pain.”* *“Personally, I have no reservations because there’s literature out there that supports the use, and from personal experience with my patients it seems to help so if it’s legal if it’s really made legal then we can go ahead and prescribe.”*		
**Subjective norm**	Sociocultural Classification of Hemp	Stigma/Acceptance of Hemp post legalization Mental health concerns for medicinal hemp Clinical non-prescription use of hemp
*“…You see there’s a big debate that marijuana causes mental health”* *“Yes, I do see concerns for mental health. You know any form of addiction is a disease…so like I said earlier the rational use of a drug is important, how you will ensure that the end users are using it right…that is going to be a problem it’s going to be a challenge.”* *“In our local context the hesitation cuts across. Even the younger age group who have tried it before will not openly accept that they have been doing it or they have used it before. They will pretend to have an aversion to it because they don’t want to seem like a bad person. Because in the general public eye it is not acceptable.”* *“The general hesitancy to even try medicinal cannabis is due to lack of knowledge. And lack of education”*		
**Perceived Behavioral Control**	Control and management Adverse effects	Knowledge and perception of legislative structures Regulation of medicinal hemp
*“Currently there is no clinical policy or guidelines. It is not available in our national guidelines, so this use is on an individual basis, and we have found experience with it…* *“So far in terms of substances that have a potential of abuse, most of the time an effective management is to limit the people that can prescribe it such that only people who clearly know how to use and prescribe it are allowed to prescribe it and also limit the number of places that it is available, make sure that the places are licensed; somewhere that they can’t go over the counter to buy from some random pharmacy and that the places know what they are selling, who they are selling to and for what indication.”* *“Like any other drug they need to make sure the indications are clear, which level prescriptions should come from are clear and of course liaise with pharmacies so that they can’t be leaked and purchased for other purposes because that’s the problem.”* *“If we regulate it we can get a better medicinal benefit out of it. To harness its full potential, we have to regulate it.”* *“… it is very easy to get opioids in this country. Some pharmacists will not sell, others will sell because they are looking for money and don’t care whether there’s the indication or not. Even though it’s not supposed to be sold over the counter people go over the counter and buy tramadol…so unless we put in place measures that will limit let’s say the production, distribution, storage and medical application it’s going to be like the challenge we have with opioids”*		
**Behavioral Intention**	Policy Landscape	Legislation shift of Medicinal hemp Inclusion of medicinal hemp in medical practice
“*It’s possible have medical advancement in the future towards the use of cannabis because in various countries it’s being legalized medically and of course everything comes with local research so if trials are confirmed locally and its efficacy in our population is saturated then it’s easier so I think it would be driven by research.”* *“Why not, anything that will help our patients and ourselves…there are drugs in use that are worse off even in their therapeutic use than cannabis but is being used in conventional medicine practice. It’s about having the right indications and right policies.”* *“I am not averse to prescribing it if it’s legal and made clear, I will prescribe it but not right from the onset.”*		

Fourteen out of the 20 respondents were male and 6 were female. Among these participants, 20% were residents or consultants in anaesthesiology and pain management, 25% in oncology, 30% in neurology and 25% in orthopaedics. Ninety-five percent of the medical doctors interviewed were trained in Ghana and 70% were practising in government hospitals. All the respondents practiced in tertiary and specialist hospitals in Ghana. Sixty percent of these practitioners had between 5–10 years in practice, whereas the remaining 40% had 11 – 20 years in practice. Only one (1) doctor reported personal experience with prescribing medical cannabis for patients. Two (2) doctors observed other doctors using it in practice. The remaining seventeen (17) physicians had no direct experience with the use of cannabis in clinical therapy.

## Major themes (perspectives)

- Clinical Use/ Indication

Clinicians exhibited fair knowledge about the therapeutic potential of medicinal hemp. However, the category of responses could be broadly described as hearsay benefits and evidence based. The majority of the specialists attributed the clinical relevance of cannabis to evidence from the literature. Among the clinicians involved, neurologists expressed the strongest belief in the therapeutic benefits of cannabis and exhibited a relatively high level of comfort in prescribing it for their patients.

*“Personally of course I have used it for especially my dementia patients with all sorts of problems with sleep, calming down agitation and all of that, yes, we have used and then personally a few patients with severe chronic pain, but my experience mostly has been with old patients with dementia. They tend to respond favorably” (Specialist 4, Neurology).*

*“I use medicinal cannabis for our patients, some with retractable epilepsy, drug resistant epilepsy, and some with childhood epilepsy. For patients with multiple sclerosis, for pain/spasticity, chronic fatigue syndrome and bladder dysfunction we used the cannabinoid oil. (Specialist 1, Neurology).*

However, a few other doctors described them as hearsay with no clear-cut evidence.

*“I don’t know, I just feel like cannabis can only be recreational with no clinical relevance. It’s perceived to be medicinal just because it can blind people to how they truly feel”. (Resident 2, Oncology).*

- Stigma (Sociocultural Classification of Cannabis)

Clinicians communicated strong concerns about the perceptions of others in regard to the clinical adoption of medicinal cannabis. They highlighted the strong negative connotations attached to the use and recommendation of cannabis. These negative sociocultural perceptions were limited not only to fellow prescribers but also to their patients. For the patients, the recent tramadol crisis heightened in the media was reported to have accounted for their perceptions. These persons refused to use or take tramadol on the basis of perceived harm/danger.

*“The question is, will the patients be willing to start treatment if they have been prescribed cannabis? Second, will they be willing to discuss with their partners or other people who will be involved in their treatment? These are things most neurologists will be worried about rather than efficacy as for efficacy once science has proven, it is accepted. Think about what happened with tramadol, everyone was taking tramadol without any problems then suddenly patients tell you, no doctor I don’t want tramadol.” (Specialist 3, Neurology).*

*I think there will be a hesitation to its adoption into our healthcare structure because it is associated with drug abuse. So, when we ask in a triage, ‘do you take cannabis?’ They get very defensive as if it is a bad thing. (Specialist 7, Anaesthesiology).*

*“I have been here since 2015 and have personally never prescribed anything of that sort and I haven’t seen anyone prescribe anything of that sort. Therefore, we do not talk about cannabinoids here at all.” (Specialist 8, Oncology).*

### Clinical non-prescription use of cannabis

The interviewees (prescribers) acknowledged the clinical use of medicinal hemp by some patients without a direct recommendation from them. The patients often used them for the management of pain. Most of the prescribers expressed comfort in the personal clinical use of cannabis by the patient but refused to have any part in the recommendation, monitoring, or sourcing of the drug. Some prescribers also resorted to indirect means of suggesting the use of medicinal hemp. Among the interviewees, two clinicians were comfortable with the direct proposition of medicinal hemp to their patients.

Yes, I have had patients get on to medicinal cannabis use without my consent and I say okay why not? (Specialist 4, Neurology)

*Our local setting to the best of my knowledge cannabis is illegal. But I can tell you I know of people who have sourced medicinal cannabis from outside and are using it for clinical indications. (Specialist 5, Anaesthesiology).*

*“I have had other patients who come in and ask me about it. They will say they have the oil and they will swear by it that when they take it it really helps to control the pain. (Specialist 8, Oncology).*

### Policy landscape

Clinicians described the current policy landscape in Ghana as unwelcoming to the clinical use and recommendation of medicinal hemp. The current law on narcotic use does not encourage scientific research or discussions on the possible clinical role of cannabis in pharmacotherapy. Most prescribers had a blanket opinion about hemp use. “*It is illegal in Ghana!*” Despite this policy characterization, some prescribers outlined the need for more cannabis-related policies in treatment guidelines and clinical research. These responses were common amongst oncologists and neurologists. When asked about the need to incorporate medicinal cannabis into our medical system?” A prescriber answered vehemently, *“Why not? Anything that will help our patients and ourselves! There are drugs in use that are worse off; even in their therapeutic use, they are worse off than cannabis but it is being used in conventional medicine practice. It’s about having the right indications and right policies.” (Specialist 4, Neurology).*

Considering the scope of cancer therapy there is a need to incorporate medicinal cannabis. The more options patients have the better. (Specialist 3, Neurology)

*100%. There is a critical need to include it in the clinical setting. This is the oncology center, so we need it. I am not averse to prescribing it if it’s legal (Specialist 8, Oncology).*

### Adverse effects

The interviewees also raised concerns about the profile of adverse effects associated with the use of medicinal cannabis. The principal concern was the effect of cannabis on mental health. However, most respondents maintained that the effective and controlled use of cannabis was unlikely to elicit any mental health concerns. Some practitioners also attributed the potential clinical value of cannabis to its euphoric elements. In their opinion, patients tend to feel better because of the false perception of reality. The general apprehension about the adverse effects of cannabis was mostly linked with recreational use.

Additionally, other clinicians were worried about the indirect endorsement of recreational cannabis by the clinical adoption of medicinal hemp.

*Medical adoption can endorse recreational use on the basis of our culture in Ghana. People can tell you ‘Oh I am taking it because my doctor prescribed this for me’.” (Specialist 3, Neurology).*

*Medicinal cannabis has been found not to cause as much euphoria for addiction and mental health problems that has been associated with cannabis itself. I will not be so worried about its untoward mental health implications (Specialist 5, Anaesthesiology).*

*I will be concerned about mental health and if I will be strangled in the consultation room. (Resident 2, Oncology)*

### Control and Management

With respect to the control of medicinal hemp, the clinicians refer to the weak regulatory system in the country and the need for more robust structures. As an example, most of the interviewees highlighted the surge in tramadol abuse facilitated by a weak legitimate supply chain for opioids.

The whole problem is our system of drugs entering the country. Just like tramadol…gaps in the system. (Specialist 4, Neurology)

*It is very easy to obtain opioids in this country. Some pharmacists will not sell, others will sell because they are looking for money and do not care whether there is an indication or not. Even though it is not supposed to be sold over the counter, people go over the counter and buy tramadol. Therefore, unless we put in place measures that will limit let’s say the production, distribution, storage, and medical application it is going to be like the challenge we have with opioids. (Specialist 6, Anaesthesiology).*

*In the larger context of control, that is where some of us have a little bit of hesitance because even with our opioids there is absolutely no control. (Specialist 5, Anaesthesiology).*

## Discussion

The medicinal utilization of hemp has always been an issue of great controversy among healthcare workers around the world (Irvine 2023). Beyond scientific knowledge, the value of medicinal hemp use differs amongst stakeholder groups such as general practitioners, specialists, and patients (Newhart and Dolphin 2023).

Most clinicians are fairly knowledgeable about the medicinal potential of hemp but the extent of knowledge and the quest for more information vary among clinical specialties (Gardiner et al. 2023; Mathern et al. 2023; Crowley et al. 2023)).

The study participants acknowledged the medicinal potential of hemp and outlined multiple indications as recorded in the literature. This notwithstanding, most of these prescribers expressed doubts about the clinical relevance of hemp. This contradiction in knowledge and perceptions among physicians was captured by Zolotov et al. (Zolotov et al. 2018).

In this study, neurologists demonstrated the greatest interest in the clinical value of hemp which can be attributed to the frequent association between the clinical indications for cannabis and the field of neurology (Treister-Goltzman et al. 2000). Generally, physicians are more likely to exhibit support for medicinal cannabis if they are in a specialty field rather than in general practice (Gardiner et al. 2023).

The study also sought to explore the subjective norms associated with a prescriber’s intention to prescribe or consider medicinal hemp. Consistent with the literature, social stigma strongly influences the prescribers’ perceptions of medicinal cannabis (Szaflarski et al. 2020).

Cannabis use is still viewed as an aberrant activity because of its popular description as a recreational drug and its common ties with crime (Szaflarski et al. 2020), (Bottorff et al. 2023).

Among clinicians and the hospital workforce, the stigma is associated primarily with the illicit status of cannabis (Isaac et al. 2023). The ideology that medicinal cannabis is unconventional and falls short of the standards of biomedicine remains a bone of contention among physicians (Zolotov et al. 2018; Rønne et al. 2023; Hout et al. 2023). Generally, physicians are known to feel much more at ease in providing medicinal cannabis when its considered legal within their jurisdiction of practice (Yeroushalmi et al. 2020).

Contrary to the opinions of the interviewees, the existing policy framework of the Ghana Food and Drugs Authority makes provision for the clinical prescription of locally unregistered medicines such as cannabis (Parliament of Ghana 2012).

Despite this inherent policy flexibility to prescribe hemp when needed, the overarching narcotic laws in Ghana have shrouded clinicians’ beliefs about the degree of control they possess on their decision to prescribe or recommend medicinal hemp.

The majority of the study participants described the medicinal use of hemp as illegal in Ghana hence categorized its medical recommendation as outside their voluntary control. As posited by the theory of planned behaviour, the lack thereof of a perceived ease in recommending medicinal cannabis exerts a direct effect on the prescribing behaviour of the clinician (Trafimow et al. 2023), (Kiriakidis 2023).

Stigma with medicinal hemp also tends to be a negative component of the physician–patient relationship (Ryan et al. 2023; Ryan and Sharts-Hopko 2023). Cannabis use is viewed through a sociocultural lens of illicit drug use, and it creates a barrier to physician engagement on this subject (Ryan et al. 2023; Stigma 2023). In South Africa, physicians attributed their discomfiture with cannabis use to the negative or exclusionary position of medical institutions towards cannabis use in medical treatment (Audain 2024) Similar trends were realized in Ghana with the media portrayals of the tramadol crisis as a moral catastrophe (Korem and Id 2023).

With respect to the potential adverse effects of medicinal cannabis, much concern is always attached to the possible diversion from licit to illicit use (Rønne et al. 2023; Hout et al. 2023; Ziemianski et al. 2023)).

This fear that cannabis would be acquired ‘medicinally’ as a valid façade for illicit use has always been a matter of major concern for health professionals (Gardiner et al. 2023).

However, such concerns are not exclusive to medicinal cannabis. All drugs one way or another pose different adverse challenges and the remaining question is, “does the risk outweigh the benefit?” (Ryan and Sharts-Hopko 2023). A study conducted in America reported that approximately 90% of palliative care providers deemed cannabis useful in the treatment of pain, nausea, and appetite loss, whereas more than 50% believed the adverse effect profile was the same or less problematic than conventional alternatives (Luba et al. 2023). In Africa, doctors and pharmacists characterised cannabis as problematic owing to knowledge gaps among prescribers, social barriers, and religion (Kitchen et al. 2024b).

Similar to previous findings in literature, there was general support for medicinal hemp endorsement, but it varied according to medical specialty and practice settings (Gardiner et al. 2023). The clinicians with experience in prescribing medicinal hemp exhibit more certainty with its clinical efficacy and less anxiety with the possible adverse effects (Rønne et al. 2024), (Rønne et al. 2023).

Overall, facilitating favourable attitudes in terms of knowledge acquisition on medicinal hemp and encouraging a more enabling policy environment devoid of stigma exerts a direct effect on a prescriber’s intention to consider hemp in clinical therapy.

### Limitations

The study setting in urban Accra and the diversity of medical specialties employed in the study population are limitations of this study. The research was constrained to specialties that addressed indications known to have some level of benefit when managed with medicinal hemp.

The study setting solely within the capital city did not reflect the possible regional differences among clinicians in Ghana. Considering the broad legislative underpinnings of medicinal hemp, future research should include other healthcare worker cadres, non-urban settings, and the opinions of key stakeholders such as policy actors and regulators.

## Conclusion

The prescribers’ intention to recommend medicinal hemp is influenced by a complex interplay of various socio-political factors such as knowledge, policy, cultural environment, and stigma.

This study not only highlights the perspectives of physicians but situates the associated factors within a theoretical framework that captures a thematic approach to future policy development related to medicinal hemp.

## Supplementary Information

Supplementary Material 1.

Supplementary Material 2.

## Data Availability

The datasets used and/or analyzed during this particular study are available from the corresponding author upon reasonable request.

## Abbreviations

GP: General Practitioner
THC: Tetrahydrocannabinol
CBD: Cannabidiol
TPB: Theory of Planned Behavior
