# Supplementary material for: Utilization of medicinal hemp: a qualitative analysis of clinicians’ perspectives in Ghana
Source: J Cannabis Res. 2025 Mar 17;7:16. doi: 10.1186/s42238-025-00271-1 (PMC11912765; doi:10.1186/s42238-025-00271-1)
Supplement: Supplementary file 1 — Supplementary Material 1. [file 42238_2025_271_MOESM1_ESM.docx]

**APPENDIX I**

PARTICIPANT INFORMATION

**Title:** Legalization of medicinal cannabis in Ghana: perspectives of health care practitioners

**Name of Principal Investigator:** Anoa Aidoo

**Address:** School of Pharmacy, University of Ghana, P. O. Box LG 43, Legon Accra, Ghana.

**Telephone number:** +233209061866

**Email:** [anoaaidoo@live.com](mailto:anoaaidoo@live.com)

**General Information about Research**

Cannabis has been categorized as an illegal substance due to adverse effects associated with its use. However, cannabis also has a number of important clinical indications. This study seeks to obtain information on the knowledge and perception of medical practitioners on the therapeutic use and legalization of medicinal cannabis in Ghana.

**Possible Risks and Discomforts**

This study shall pose no foreseeable risks or harm (physically or psychologically) to respondents.

**Possible Benefits**

This study does not present any direct personal benefit to participants. However, it will yield substantive information which may influence future policies.

**Confidentiality**

All study participants shall remain anonymous and all information provided shall be kept confidential. The data would be analyzed in such a manner that anonymizes all respondents.

**Compensation**

Participation will be voluntary and will not attract any compensation

**Voluntary Participation and Right to Leave the Research**

Participation in this study will be voluntary and respondents shall have the right to decline participation at any time during interviews if they wish.

**Contacts for Additional Information**

Anoa Aidoo (Principal Investigator)

University of Ghana, School of Pharmacy

Tel: 0209061866

Email: [anoaaidoo@live.com](mailto:anoaaidoo@live.com)

**Your rights as a Participant**

This research has been reviewed and approved by the University of Ghana School of Pharmacy (UGSOPEC). If you have any questions about your rights as a research participant, you can contact the Administrative Assistant at the General Office between the hours of 8:00 am and 4:30 p.m. through the phone line **0272244321** or email address: [ugsopec@ug.edu.gh](mailto:ugsopec@ug.edu.gh) .
