# Supplementary material for: Utilization of medicinal hemp: a qualitative analysis of clinicians’ perspectives in Ghana
Source: J Cannabis Res. 2025 Mar 17;7:16. doi: 10.1186/s42238-025-00271-1 (PMC11912765; doi:10.1186/s42238-025-00271-1)
Supplement: Supplementary file 2 — Supplementary Material 2. [file 42238_2025_271_MOESM2_ESM.docx]

**Appendix III**

**INTERVIEW GUIDE**

Introduction: My research seeks to explore the views of physicians on the use of medicinal cannabis and its legalization in Ghana. This will be an interview that will take about 15 minutes of your time.

**Personal information**

Can you kindly provide me with these details?

1. Sex
2. Age range (<25, 25-35, 36-45, 45-55, >55)
3. Where did you have your medical training?
4. What is your practice area/Specialty?
5. Which level of care are you currently providing (primary, secondary or tertiary)
6. How many years have you practiced as a physician?
7. How many years have you practiced in Ghana? (if different; where else have you practiced)

**Knowledge on medicinal cannabis**

1. In your opinion what is medical cannabis?
2. Are there disease conditions requiring the use of medical cannabis?
3. Can you tell me about the clinical efficacy of cannabis for the aforementioned indications?
4. Are there any alternative treatment options aside cannabis in these instances; and how do they compare with cannabis in terms of efficacy?

**Medicinal cannabis use in Ghana**

1. In your opinion has there been a change in cannabis legislation around the world? Can you tell me about it?
2. Has Ghana experienced any change in legislation over the past five years with respect to use of medicinal cannabis?
3. Considering the scope of medical practice in Ghana, is there a need to include medicinal cannabis as part of therapy? Please explain further
4. What will inform your recommendation of medicinal cannabis for a patient?

**Knowledge and perception of legislative structures**

1. Are there any clinical guidelines or policies outlining medicinal cannabis use in Ghana? (if yes, which?)
2. Has the recent legalization of medicinal cannabis affected clinical practice in Ghana? Please explain
3. Are there any concerns for mental health with the use of medicinal cannabis? Please explain
4. How do you think medicinal cannabis should be managed in Ghana (cultivation, local industries, capacity building)?
5. Has legalization of cannabis affected acceptance of its use?
